# Comparative Proteome Signatures of Trace Samples by Multiplexed Data-Independent Acquisition

**DOI:** 10.1016/j.mcpro.2021.100177

**Published:** 2021-11-15

**Authors:** Claudia Ctortecka, Gabriela Krššáková, Karel Stejskal, Josef M. Penninger, Sasha Mendjan, Karl Mechtler, Johannes Stadlmann

**Affiliations:** 1 Research Institute of Molecular Pathology (IMP), Vienna BioCenter (VBC), Vienna, Austria; 2 Institute of Molecular Biotechnology of the Austrian Academy of Sciences (IMBA), Vienna BioCenter (VBC), Vienna, Austria; 3 The Gregor Mendel Institute of Molecular Plant Biology of the Austrian Academy of Sciences (GMI), Vienna BioCenter (VBC), Vienna, Austria; 4 Department of Medical Genetics, Life Sciences Institute, University of British Columbia, Vancouver Campus, Vancouver, British Columbia, Canada; 5 Institute of Biochemistry, Department of Chemistry, University of Natural Resources and Life Sciences (BOKU), Vienna, Austria

**Keywords:** data-independent acquisition, isobaric multiplexing, large sample cohorts, identification-free data aggregation, ultralow peptide input, AGC, automatic gain control, DDA, data-dependent acquisition, DIA, data-independent acquisition, NCE, normalized collision energy, PSM, peptide-to-spectrum match, RI, reporter ion, RT, retention time, TMT, tandem-mass-tag

## Abstract

Single-cell transcriptomics has revolutionized our understanding of basic biology and disease. Since transcript levels often do not correlate with protein expression, it is crucial to complement transcriptomics approaches with proteome analyses at single-cell resolution. Despite continuous technological improvements in sensitivity, mass-spectrometry-based single-cell proteomics ultimately faces the challenge of reproducibly comparing the protein expression profiles of thousands of individual cells. Here, we combine two *hitherto* opposing analytical strategies, DIA and Tandem-Mass-Tag (TMT)-multiplexing, to generate highly reproducible, quantitative proteome signatures from ultralow input samples. We developed a novel, identification-independent proteomics data-analysis pipeline that allows to quantitatively compare DIA-TMT proteome signatures across hundreds of samples independent of their biological origin to identify cell types and single protein knockouts. These proteome signatures overcome the need to impute quantitative data due to accumulating detrimental amounts of missing data in standard multibatch TMT experiments. We validate our approach using integrative data analysis of different human cell lines and standard database searches for knockouts of defined proteins. Our data establish a novel and reproducible approach to markedly expand the numbers of proteins one detects from ultralow input samples.

## Highlights

• DIA-TMT provides reproducible, quantitative proteome signatures at high throughput.
• Proteome signature inferred cell type characterization is highly accurate.
• Proteome signatures accurately highlight underrepresented cell types.
• ID-independent DIA-TMT is more reproducible than standard DDA acquisition strategies.

## Graphical Abstract

**Figure:**
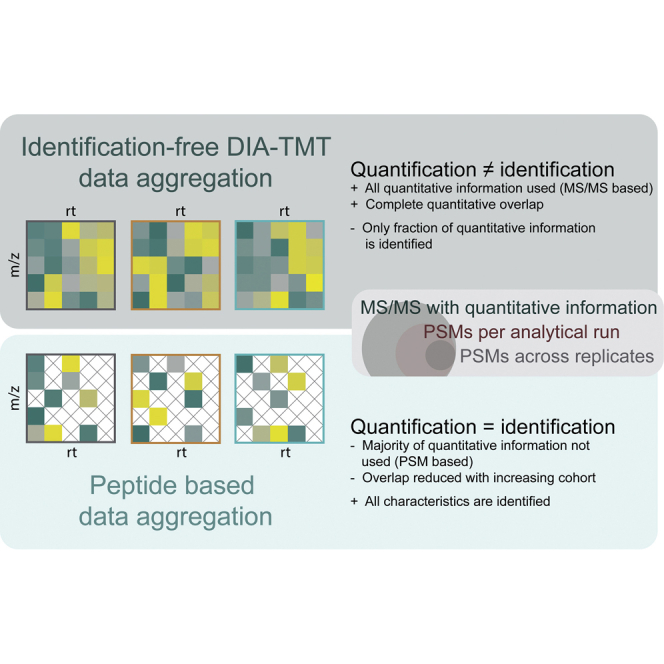

## In Brief

Proteomics faces the challenge of reproducibly comparing the protein expression profiles across large sample cohorts. Here, we combined two *hitherto* opposing analytical strategies, DIA and isobaric labeling to generate highly reproducible, quantitative “proteome signatures”. These signatures decouple peptide identification from quantification to quantitatively compare hundreds of samples. DIA-TMT data provides complete quantitative signatures independent of peptide identification that distinguish cell types down to single protein knockouts in high-throughput even at ultralow input.

Single-cell proteomics aims at assessing protein expression within individual cells with far-reaching opportunities for a better understanding of fundamental biology or disease states. Currently, protein analysis at single-cell resolution is still largely antibody based, therefore relying on the availability of such. This not only greatly limits the throughput of these techniques, but also requires preformed hypotheses (*i.e.*, flow cytometry and mass cytometry). At present, mass-spectrometry-based proteomics is the only viable technology for discovery and hypothesis-free protein analysis.

While the comprehensive proteomic characterization of individual mammalian cells is still limited by the sensitivity of current MS/MS-based workflows, the concept of multiplexed shotgun proteomics analyses of individual cells in conjunction with a highly abundant, congruent carrier proteome has been seminal to the field (1). The use of established *in vitro* stable-isotope labeling techniques (*e.g.*, TMT) not only increases precursor- and fragment-ion abundances for peptide identification and quantification from ultralow input samples, but also increases sample throughput. Currently, such multiplex single-cell proteomics workflows have allowed for the quantitative analysis of up to 13 barcoded single cells in one analytical run (2).

Nevertheless, paralleling state-of-the-art transcriptomic datasets, single-cell proteomics ultimately faces the challenge to comparatively analyze hundreds or even thousands of ultralow input proteomics samples (3, 4, 5). Such sample sizes vastly exceed the capacities of any currently available MS multiplexing technology (6). Merging large numbers of individual quantitative shotgun proteomics files into one dataset often entails that a considerable number of peptides are not reliably identified in all analytical runs (7). This method-intrinsic accumulation of “missing values” greatly limits the use of such data-dependent acquisition (DDA) strategies for the comparative analysis of protein levels in large sample numbers, as are necessary for reproducible single-cell proteomics, which is currently addressed by various computational data imputation or “match-between runs” methods (8, 9, 10).

By contrast, data-independent acquisition (DIA) regimes, which subject all precursor ions within a defined m/z window to MS/MS analysis, have been shown to allow for the robust quantification of protein expression, even across extremely large sample cohorts (11). Recently, DIA strategies were further extended to sequentially windowed DIA schemes (SWATH), specifically designed to cover all theoretical mass spectra and to thereby provide deep proteome coverage (12, 13). To develop a scalable high-throughput data-acquisition strategy for comparative single-cell proteomics, we combined *in vitro* multiplexing strategies for MS/MS-based quantification (*i.e.*, TMT10-plex Isobaric Label Reagent Set) and small window DIA data-acquisition regimes (*i.e.*, m/z = 6 Th) for the analysis of ultralow protein amounts.

## Experimental Procedures

### Sample Preparation

Tryptic digests were obtained from Promega (K562, catalogue number: V6951) and Thermo Fisher (HeLa, catalogue number: 88328) and were TMT10-plex-labeled according to manufacturer’s instructions. Briefly, samples were labeled in 100 mM TEAB and 10% ACN for 1 h at room temperature and subsequently quenched with 5% hydroxylamine/HCl for 20 min at room temperature and subsequently mixed corresponding to each TMT10 plex. To exclude any label specific effects, three mixes were compiled as follows: Mix 1: K562 cell lysate channels: 126, 127N, 127C, 128N, 128C – HeLa cell lysate channels: 129N, 129C, 130N, 130C, 131; Mix 2: inverted Mix 1; Mix 3: K562 cell lysate channels 126, 127C, 128C, 129C, 130C - HeLa cell lysate channels: 127N, 128N, 129N, 130N, 131. Prelabeled Pierce TMT11-plex Yeast Digest Standard (catalogue number: A40938) was resuspended in 0.1% TFA and diluted to 0.5, 1, 5 and 10 ng total peptide input.

### LC MS/MS Analysis

Samples were measured on an Orbitrap Exploris 480 Mass Spectrometer (Thermo Fisher Scientific) with a Dionex UltiMate 3000 high-performance liquid chromatography RSLCnano System (Thermo Fisher Scientific) coupled *via* a Nanospray Flex ion source (Thermo Fisher Scientific) equipped with FAIMS Pro (Thermo Fisher Scientific). Reversed-phase chromatographic separation was performed on a μPAC (50 cm, PharmaFluidics) column or nanoEase M/Z Peptide BEH C18 Column (130 Å, 1.7 μm, 75 μm × 150 mm, Waters) developing a two-step solvent gradient ranging from 2 to 20% over 47 min and from 20 to 32% ACN in 0.08% formic acid within 15 min, at a flow rate of 250 nl/min.

For both, DIA and DDA experiments, the FAIMS Pro device was constantly operated at a compensation voltage of −50. In DDA LC-MS/MS experiments, full MS data were acquired in the range of 370 to 1200 m/z at 120,000 resolution. The maximum automatic gain control (AGC) and injection time were set to 3e6 and automatic maximum injection time. Multiply charged precursor ions (2–5) were isolated for higher-energy collisional dissociation MS/MS using a 2 Th wide isolation window and were accumulated until they either reached an AGC target value of 2e5 or a maximum injection time of 118 ms. MS/MS data were generated with a normalized collision energy (NCE) of 34, at a resolution of 60,000, with the first mass fixed to 100 m/z. Upon fragmentation precursor ions were dynamically excluded for 120 s after the first fragmentation event.

DIA experiments were acquired in the most densely populated precursor range of 400 to 800 m/z at a resolution of 45,000, based on multiple analytical runs of human whole cell digests. The AGC was set to 2e5 and the maximum injection time was automatically determined for each scan. DIA windows were constructed under the premise of sampling every chromatographic peak at least twice, yet limiting intentional coisolation of multiple precursors to a minimum. With an average full width at half maximum of all chromatographic peaks of 8 s, a corresponding average DIA cycle time of 8 s allowed the definition of 80 DIA windows per cycle, acquired with a 5 Th isolation windows (5 Th windows, 1 Th overlap) with stepped NCE 35, 37.5, and 45.

### Data Analysis

TMT10-plex reporter ion (RI) quantification was performed within the Proteome Discoverer environment (version 2.3.0.484) using the in-house developed, freely available PD node “IMP-Hyperplex” (pd-nodes.org) with a reporter mass tolerance of 10 ppm. The software extracts raw RI intensities from respective spectra for quantification.

Peptide identification was performed using the standard parameters in SpectroMine 2.0 against the human reference proteome sequence database (UniProt; version: 2018-11-26 accessed April 2019; 20,253 entries) and the yeast reference proteome sequence database (Uniprot; version: 2019-07-25; accessed November 2019; 6049 entries). Specific tryptic enzymatic cleavages with maximum two missed cleavages were allowed and limited to 7 to 52 amino acids per peptide. We included carbamidomethlylation on cysteine, TMT10-plex on lysine, and all N-termini as fixed modifications, while acetylation on protein N-terminal peptides and methionine oxidation were set to variable. SpectroMine by default automatically calculates the optimal mass tolerances at the MS and MS/MS levels and performs a mass calibration for each feature. Identifications are then filtered for 1% FDR on the peptide-to-spectrum match (PSM), peptide and protein group level (Supplemental Data S1–S4).

TMT10-plex spectral libraries for Spectronaut were generated from the 10 ng DDA files (HeLa/K562 including 8030 and TKO11-yeast with 6511 precursors) and adapted using a customized script, kindly provided by Oliver Bernhard from Biognosys (deposited on GitHub ctorteckac/DIA-TMT). In brief, the script adds the defined RI masses of the TMT10-plex reagents per modified peptide as additional fragment ion to each MS/MS scan. This modified library allows Spectronaut to search the DIA runs against the provided TMT library including all TMT fragment ions. Only spectra scoring above the 1% FDR cutoff as described in the SpectroMine search parameters were included into the TMT library. For the library searches, Spectronaut calculates the ideal mass tolerances similarly for library generation and spectral matching, based on extensive automated mass calibration. For this the most intense peak within the previously defined mass tolerance is selected and matched with a minimum of three matching fragment ions per MS/MS scan. Retention time (RT) referencing was performed based on the iRT Reference Strategy using Deep Learning Assisted iRT Regression with minimum R^2^ of 0.8. Decoy spectra are generated in a “mutated” manner, where the amino acid positions are scrambled, which were then used for FDR filtering of 1% on precursor and protein levels (Supplemental Data S5–S8).

**Fig. 1:**
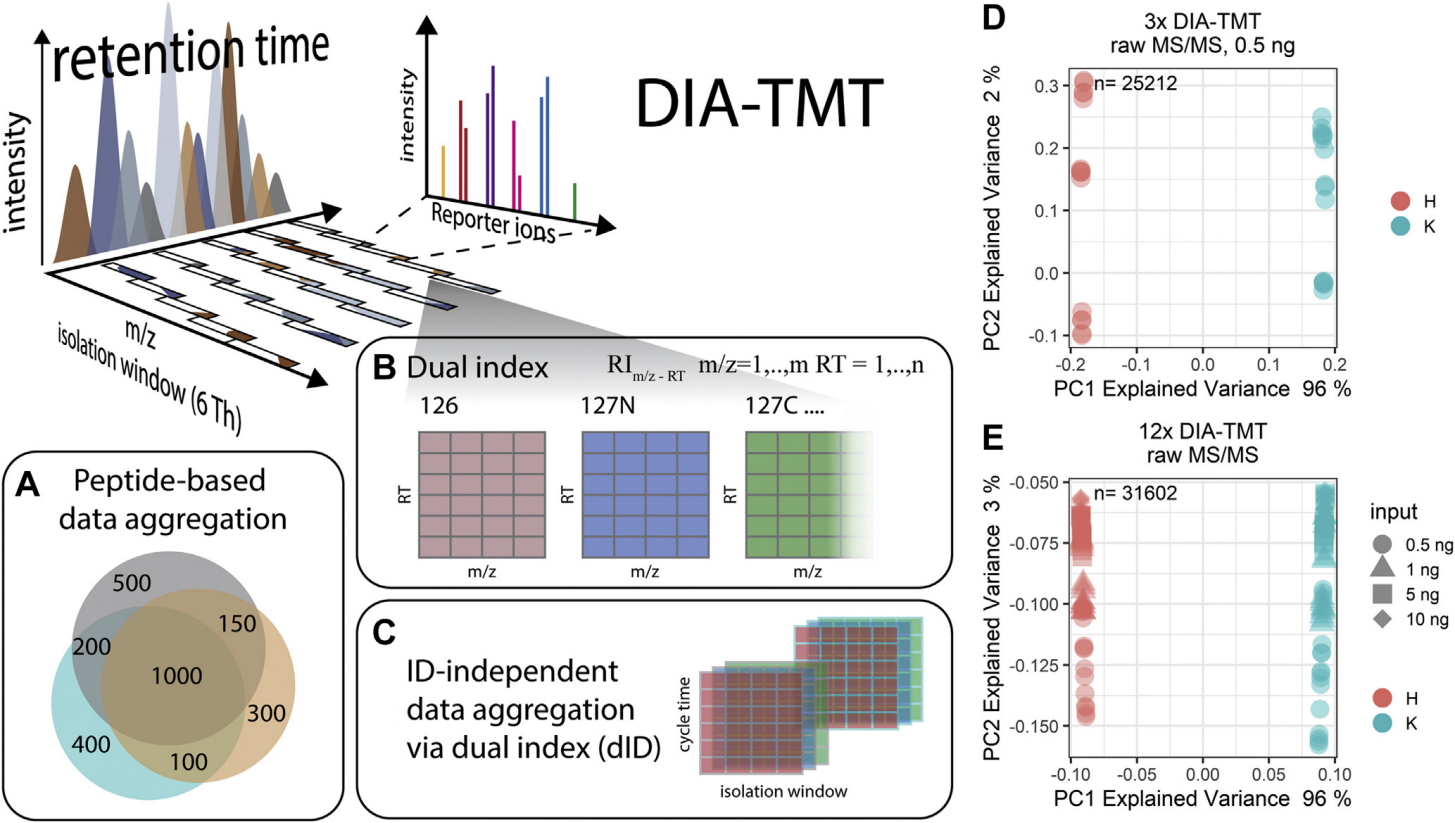
**ID-independent DIA-TMT analysis creates cell type specific clusters.***A*, peptide-based data aggregation of DIA-TMT results in a decrease overlap between replicates without computational generation of quantitative data. *B*, all MS/MS scans from the DIA-TMT files are indexed using the dual indexing (dID) = [RT_1,2,…,m_ × m/z_1,2,…,n_] according to the RT (RT 1, 2, …, m) and central mass (m/z 1, 2, …, n). In conjunction with the quantitative RI values, a grid-like 3D map or proteome signature is created, which is used for (*C*) ID-independent data aggregation theoretically resulting in a complete overlap of all MS/MS scans. PCA of (*D*) three DIA-TMT runs (30 multiplexed samples) at 0.5 ng total peptide input or (*E*) 12 DIA-TMT runs (120 multiplexed samples) at four peptide inputs (0.5, 1, 5, 10 ng) ID-independently aggregated. Samples are colored according to channel loadings and the respective peptide inputs are indicated with different *symbols*. H = HeLa cells, K = K562 cells. n, number of MSMS scans included in PCA.

Peptide-based data aggregation was performed using standard parameters *via* Spectronaut or SpectroMine for DIA or DDA, respectively (Fig. 1*A*). By default, global median normalization is performed across all experiments. RI intensities were directly imported into R for further processing. PSMs were filtered to unique peptides using the best scoring (Q-value) PSM for subsequent analysis. Venn Diagrams are based on unique peptide sequences and were calculated using BioVenn (Supplemental Data S2, S4, S6, and S8) (14).

Identification (ID)-independent RT alignment was based on the elution time points of eight doubly charged precursors (466.7743, 660.3674, 464.779, 437.2732, 587.3539, 587.864, 706.4723) observed in all runs and was performed prior to ID-independent data aggregation using a “dual indexing approach” (illustrated in Fig. 1*B*). In detail, DIA MS/MS scans were indexed according to isolation window (m/z = 1, …, m) and RT (RT = 1, …, n), resulting in a unique index (dID) per MS/MS in each analytical run.

$$\left[\begin{array}{lll} RI_{1,1} & \cdots & RI_{1,m}\\ \vdots & \ddots & \vdots \\ RI_{n,1} & \cdots & RI_{n,m} \end{array}\right]$$

Based on the dID and the prescheduled acquisition scheme, all MS/MS scans are classified into a complete matrix. Each matrix presents quantitative values of all RIs present in the scan (*i.e.*, scan Nr.: 1, dID = 1:1, quantitative values for 126, 127N, … 131N; Fig. 1*B*). Each TMT10-plex analytical run therefore gives rise to ten unique quantitative matrices based on dID, which subsequently allow to aggregate multiple analytical runs without the accumulation of missing data (Fig. 1*C*). Multibatch ID-independent datasets were normalized by scaling to equal signals per channel. If indicated, missing value imputation was performed based on random numbers from normal distribution shifted into the noise by 1.8 in log_10_ space (15). For both, peptide-based and ID-independent analysis, a ComBat-based batch correction was performed within the R environment using the sva package (16, 17).

Fragments Per Kilobase of transcript per Million mapped reads (FPKM) values were extracted directly from the publicly available ENCODE project (GEO accession: GSE33480) from HeLa and K562 datasets, from which fold changes were calculated. For both DIA and DDA data, PSMs were grouped according to their Master Protein Accession, RI intensities were averaged across replicates, and fold changes in protein expression between HeLa and K562 were calculated. Using the Uniprot database “Retrieve/ID-mapping” web interface, protein accessions were converted to gene names, which were then intersected with the transcriptome-derived FPKM fold changes. Remaining proteins/transcripts were plotted according to their transcript fold change, and the top 300 protein fold changes are indicated with the respective color. Gene names are indicated for the top 30 transcripts.

### Experimental Design and Statistical Rationale

In the present study, TMT10-plex labeled HeLa and K562 dilution mixes (channel distributions detailed in the sample preparation subsection) at 0.5, 1, 5, and 10 ng peptide input were acquired with one technical replicate per mix, three technical replicates per peptide input, and a total of 12 analytical runs (*i.e.*, 120 samples). The TKO11 yeast samples were acquired with one technical replicate per peptide input and a total of four analytical runs (*i.e.*, 44 samples). We did not acquire multiple technical replicates per peptide input to mimic lower numbers of “underrepresented” cell types in comparison to the HeLa/K562 samples. In this benchmarking study, we only compare sample dilutions and therefore do not present standard controls or biological replicates. Protein expression correlation is calculated *via* Pearson correlation with 95% confidence.

## Results

### DIA-TMT Provides Reproducible, Quantitative Proteome Signatures

We hypothesized that DIA of multiplexed ultralow input samples would overcome detrimental, DDA-inherent missing data points in multibatch TMT datasets, similarly to what has been reported previously (13, 18). To reduce precursor interference, we performed small window DIA (*i.e.*, 6 Th, detailed in Experimental Procedures) of TMT10plex-labeled tryptic digests derived from two human cell lines (*i.e.*, HeLa and K562), serially diluted to total peptide amounts similar to those expected for single mammalian cells (*i.e.*, 0.3 ng and lower) (19). Based on the prescheduled acquisition schemes of our DIA-TMT datasets, we aimed at generating comprehensive, quantitative “proteome signatures” rather than sparse peptide-identification-based profiles to detect subtle expression changes in trace samples. For this, all datasets were first RT aligned, based on the elution timepoints of eight doubly charged peptide precursor ions, evenly distributed across the entire analytical gradient and consistently detected in all samples. The RT-aligned data was then aggregated using our “dual indexing approach,” based on the central m/z of the respective isolation window and the acquisition cycle number as indices. These two characteristics (*i.e.*, m/z and RT) gave rise to unique identifiers (dID) for each MS/MS scan and, in conjunction with the sample-specific RI intensities, resulted in an abstract 3D map of the respective samples, which we refer to as “proteome signatures” (Fig. 1*B*; detailed in Experimental Procedures). Importantly, these “proteome signatures” comprise the quantification of a consistent set of *in bona fide* peptide signals across all analytical runs.

To evaluate the immediate applicability of our “proteome signatures” in an ID-independent cell type specific clustering approach, the extracted raw TMT RI intensities from all aggregated MS/MS spectra, irrespective of peptide identification (using the in-house developed PD-Node IMP-Hyperplex), were analyzed by PCA. This consistently yielded more than 25,000 datapoints from each DIA-TMT run and resulted in successful clustering of the expected cell populations, for all samples, even at 0.5 ng total protein input (Fig. 1*D*). In detail, the first principal component (PC), which is displayed on the x-axis, separates the cell lines with over 95% explained variance, while the second PC, representing only 2% of the variance, discriminates between the individual analytical runs (y-axis) (Fig. 1*D*). This confirms that the ID-independent analysis of DIA-TMT data facilitates cell-type-dependent clustering down to 50 pg peptide input per sample. Additionally, we demonstrate that the chance of coisolating two precursors in the same cycle and m/z window with exact opposing expression patterns, which would nullify the respective quantitative differences, is very unlikely.

We next assessed the scalability of our ID-independent DIA approach. To evaluate whether large sample cohorts would impact the proposed data completeness of our “proteome signatures,” we merged all 12 DIA datasets based on their dID. Intriguingly, we observed mix-independent cell type clustering, with over 95% explained variance in PC1 (Fig. 1*E*). Additionally, DIA afforded the consistent accumulation of 31,602 datapoints across all samples measured (Fig. 1*E*). This data highlights that DIA does indeed generate robust and highly congruent “proteome signatures” from larger sample sizes, even at ultralow input. Most importantly, the anticipated coisolation and cofragmentation of multiple precursors and background ions are therefore identical across analytical runs and do not result in batch effects (Fig. 1, *D* and *E*). Further, the acquisition scheme facilitates uniform sampling of the noise but capitalizes quantitative differences *via* the RI quantification. While even with small isolation windows, coisolation of multiple precursors is inevitable in DDA, the static sampling schemes in DIA result in uniform signal and noise ratios (7).

### Proteome Signatures of Single-Protein Knockouts by ID-Independent Data Aggregation

Numerous approaches aim at correcting batch effects in multibatch data sets, either pre- or postacquisition (7, 20, 21). Most importantly, however, using such statistical correction methods, peptides that were only identified in a subset of all analytical runs are extremely prone to overnormalization or exclusion. The need for such data-correction procedures thus critically limits the detection of underrepresented or unexpected cell types (*e.g.*, infiltrated tumor samples).

We therefore investigated whether the DIA strategy in conjunction with ID-independent data aggregation would allow discriminating between highly similar single protein knockout cell lines without the need for such “data correction” strategies. Therefore, we generated DIA datasets using the yeast TKO11 standard at four input levels, *i.e.*, 10, 5, 1, and 0.5 ng total peptide in technical triplicates. This commercially available TMT11-plex labeled tryptic TKO11 yeast standard, comprising three different single knockout (*met6*, *his4*, *ura2*) and wild-type yeast strains, has frequently been used for TMT benchmarking experiments (22).

**Fig. 2:**
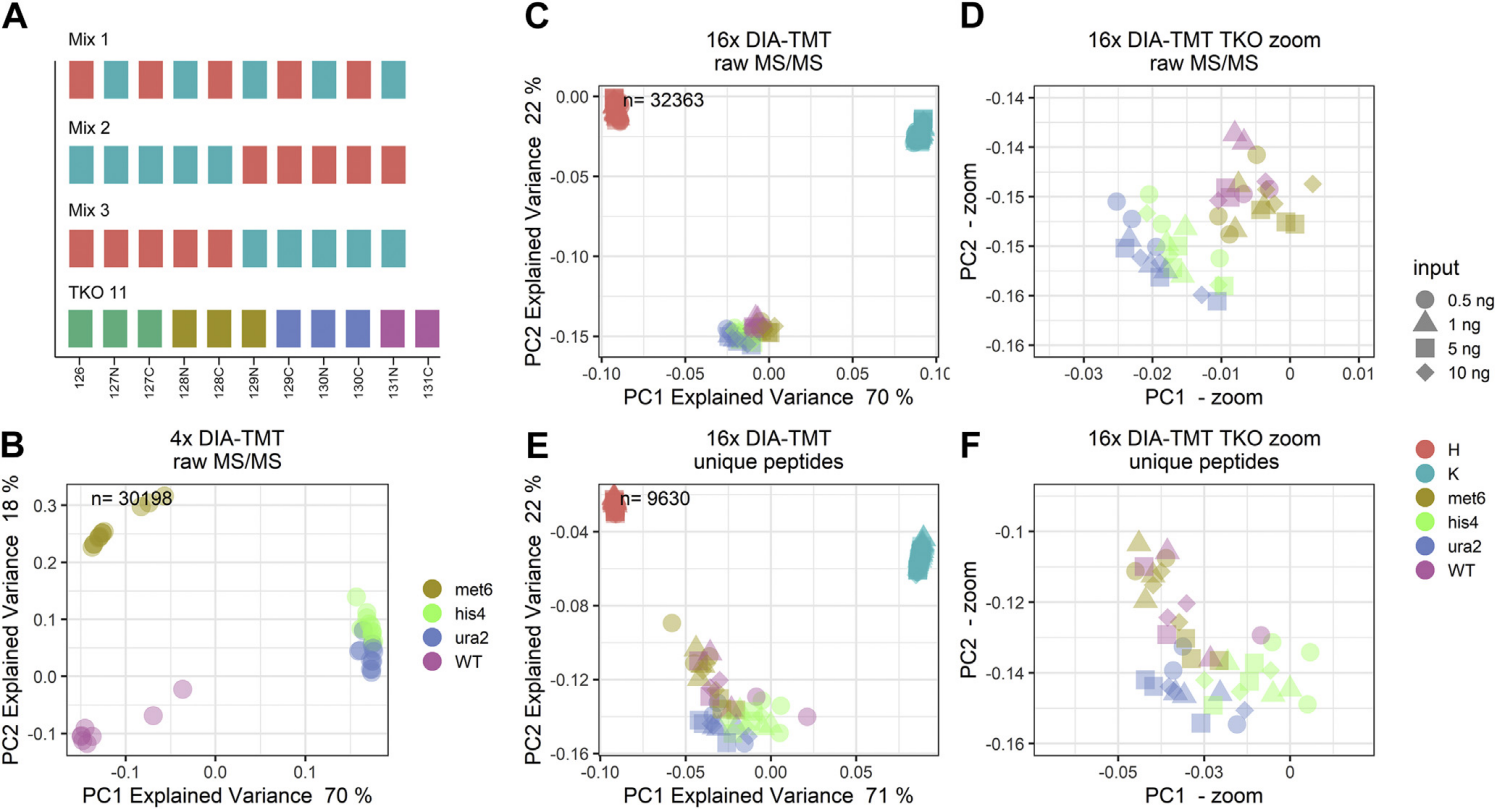
**Proteome signatures are input, batch, and species-independent across large sample cohorts.***A*, overview of isobaric labeled mixes. PCA of (*B*) four and (*C*) 16 DIA-TMT runs ID-independently aggregated at four peptide inputs (0.5, 1, 5, 10 ng). *D*, zoom into TKO11 cluster displayed in panel *C*. *E*, PCA of 16 DIA-TMT runs at four peptide inputs (0.5, 1, 5, 10 ng) with standard peptide-based aggregation and (*F*) zoom into TKO11 cluster displayed in panel *E*. Samples are colored according to channel loadings and the respective peptide input is indicated with different *symbols*. H = HeLa cells, K = K562 cells and the respective TKO11 strains (*i.e.*, WT, knockouts: *met6*, *his4*, *ura2*). n, number of unique peptides included in PCA.

**Supplemental Figure S1:**
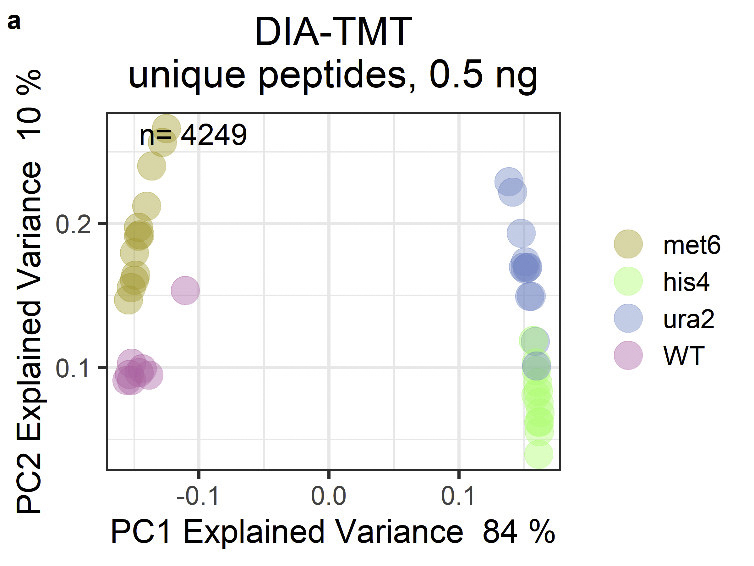
**Peptide-based DIA-TMT indicates a trend toward cell-type-dependent separation.** PCA of one DIA-TKO11 analytical run at 0.5 ng total peptide input and missing value imputation. Samples are colored according to the respective TKO11 strains (*i.e.*, WT, knockouts: *met6*, *his4*, *ura2*). n, number of unique peptides included in PCA.

While the combination of multiple analytical DIA runs of human cell lines capitalizes on their substantial biological differences (Fig. 1, *D* and *E*), the minimal disparity between the TKO11 single-protein knockout yeast strains results in a less clear separation (Fig. 2*B*). More specifically, clusters 1 and 2 only comprise the WT strain and the *met6* knockout, respectively; however, cluster 3 combines *his4* and *ura2* knockouts with a trend toward cell-type-dependent separation. Eventually, to determine the drivers of the observed cell-line clustering displayed in Figure 2*B*, we subjected the DIA TKO11 runs to a standard database search using Spectronaut. We identified met6 and ura2 proteins down to 1 ng total peptide input. We then intersected the identified MS/MS scans with the loadings of our proteome signature clustering and confirmed these scans as drivers of separation. Even though we did not identify any of the ablated proteins in the 0.5 ng DIA-TMT data, our ID-independent data aggregation strategy still allowed for cell-type-dependent clustering (supplemental Fig. S1). Most importantly, this suggests that DIA-TMT recovers relevant quantitative differences between cell types of low abundant precursors, otherwise excluded by peptide-based analysis. Additionally, our findings demonstrate that standard database searches can be used to infer hypothesis free cell type identifications to the ID-independent proteome signatures of underrepresented cell types.

### ID-Independent DIA-TMT Data Highlights Underrepresented Cell Types

Furthermore, the prospect of species- and cell-type-independent analysis would allow to postacquisitively recognize and characterize underrepresented cell types in an otherwise homogeneous dataset. To evaluate the sensitivity of our method, we merged the analogous datasets of our HeLa and K562 data with the yeast TKO11 standard. Strikingly, our ID-independent DIA data analysis retained critical cell type specific characteristics based on 32,363 quantitative MS/MS scans across 164 samples without the need for any imputation whatsoever (Fig. 2*C*). Thus, PC1 with 70% explained variance separates the main three cell types (*i.e.*, HeLa, K562, yeast), and PC2 with 22% explained variance further differentiates the two species. Of note, despite the large variance between two species and the two human cell lines, a zoom into the TKO11 cluster of ID-independent DIA data showed that we readily separate between the single yeast mutant strains (Fig. 2*D*).

To again determine the driver of the cell-type-dependent clustering, we performed standard database searching of the aggregated dual-proteome dataset using Spectronaut. The low sequence overlap between yeast and human drastically decreased complete peptide identifications to merely 16 unique peptides. We therefore performed “missing data” imputation based on commonly used Perseus parameters and aggregated all datasets (23). Missing values were replaced with random numbers from a normal distribution shifted into the noise. Based on the largely computationally generated quantitative data, yeast and human species could be separated *via* the first PC (Fig. 2*E*; with 71% explained variance). Importantly, however, separation of the individual single protein knockouts was exclusively observed in the ID-independent strategy (Fig. 2*D*). This contrasts with the peptide-based and noise-imputed data where species and cell types are successfully clustering apart (Fig. 2*E*) but single protein knockouts do not (Fig. 2*F*). Thus, our DIA acquisition scheme results in a homogeneous dataset, which can detect such small differences within highly similar and distinct samples and precludes “missing data”.

### Proteome Signature Inferred Cell Type Characterization Is Highly Accurate

**Fig. 3:**
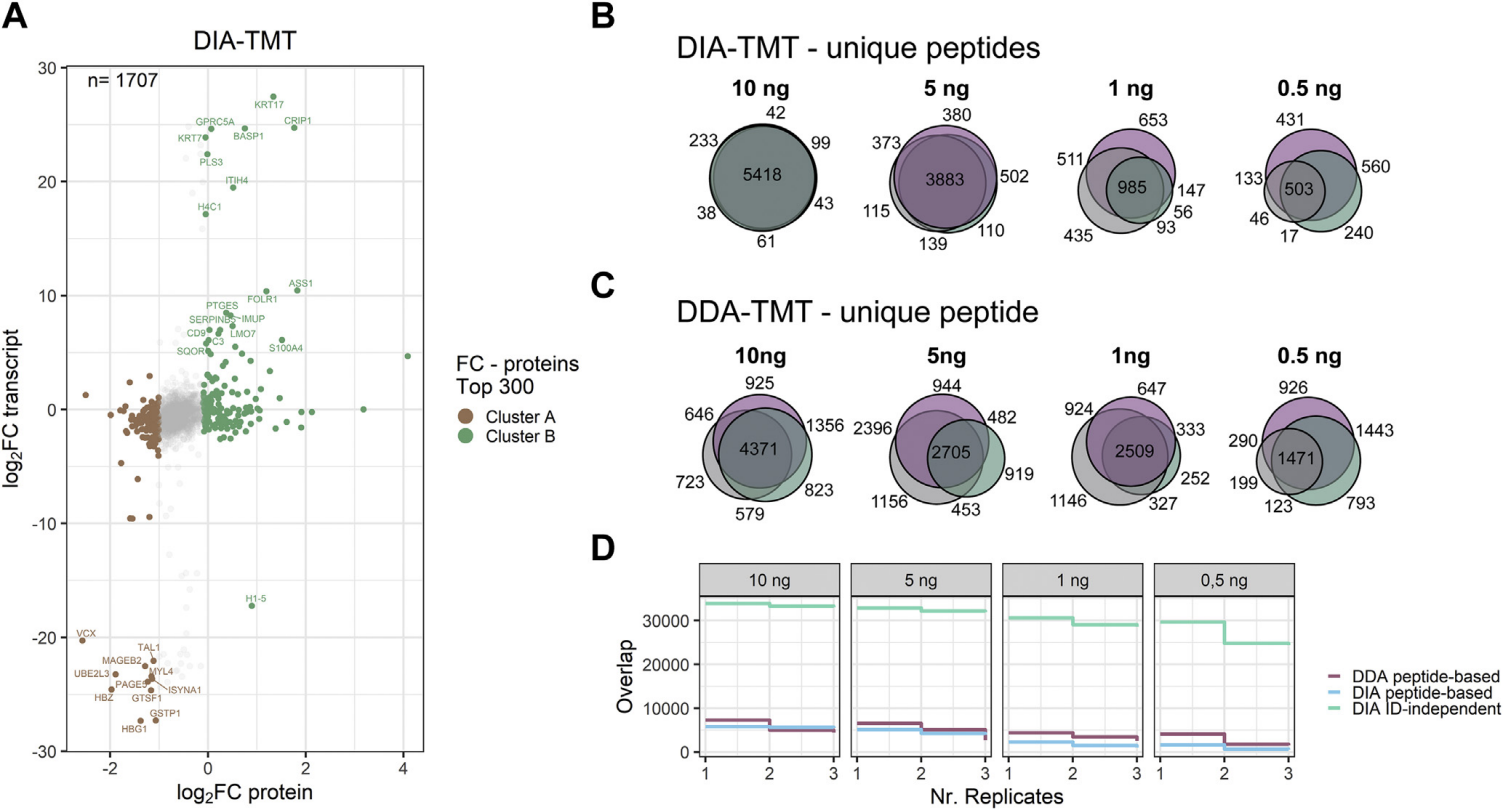
**DIA-TMT accurately reflects cell type specifics with increased replicate overlap.***A*, intersection of transcriptome (FPKM) with DIA proteome data, top 300 and 60 proteins are colored according to cluster contributions or labeled, respectively. Venn Diagrams of unique peptide sequences identified across three technical replicates at indicated peptide input of (*B*) DIA and (*C*) DDA data (mix 1 = *purple*, mix 2 = *gray*, mix 3 = *blue*). *D*, peptide-based DDA (*purple*) and DIA (*blue*) or ID-independent DIA (turquoise) accumulation of nonoverlapping peptides or indexed MS/MS scans, respectively across 12 analytical runs at decreasing peptide input (*i.e.*, 0.5, 1, 5, 10 ng).

To validate that our workflow faithfully distinguishes cell types (*i.e.*, HeLa and K562), we reanalyzed the merged data sets using Spectronaut, projected the resulting peptide identifications onto the data tables and calculated fold changes in protein abundance between clusters A and B (displayed in Fig. 1*E*). This data analysis allowed us to compare the protein expression levels of 1741 proteins across the 12 analytical runs. To align differential protein expression to transcriptome data, we then calculated fold changes of FPKM values of HeLa and K562 cells, which are publicly available *via* the ENCODE project (GEO accession: GSE33480) (24). Using the Uniprot database, Ensembl GeneIDs from the transcript data and Protein Accession numbers from Spectronaut, we mapped the expressed genes to our proteomics analysis (1707 proteins/transcripts). Transcript levels of HeLa and K562 were plotted against protein expression; the top 300 protein fold changes of cluster A and B shown in Figure 1*E*. We observed reduced protein fold changes as compared with the transcriptomics data (protein-level: ranging from −2.5 to 4.1, transcript-level ranging from −27.3 to 27.4 in log_2_ space), as expected (Fig. 3*A*) (25). Importantly, however, fold changes of the transcriptomics results paralleled our proteomics data, confirming that our ID-independent data analysis approach can indeed identify cell type specific clusters (Fig. 3*A*). This suggests that our proteome signatures allow for robust clustering and discrimination of cell types, while database reanalysis of these clusters reveals their cellular identity.

### DIA-TMT Identifications Are More Reproducible Than Standard Acquisition Strategies

Next, we evaluated the reproducibility of DIA-derived peptide identifications in ultralow input data. For this, we only considered peptides that were repeatedly identified in all three technical replicates at a given total peptide input level. As expected, at 10 ng total peptide input, we observed that DIA indeed provides highly consistent peptide identifications across multiple analytical runs (*i.e.*, >90% central overlap) (Fig. 3*B*). Importantly, this key benefit of DIA strategies was gradually lost with decreasing peptide input, presumably because of decreasing total ion current (Fig. 3*B*). Such reduction in peptide identification overlaps within replicates we expected to observe from stochastic DDA, but not DIA data. To determine whether this was sample intrinsic or indeed more pronounced in standard DDA strategies, we generated analogous datasets of HeLa and K562 mixes or TKO11 yeast samples at indicated total peptide input (*i.e.*, 0.5, 1, 5, 10 ng). We subjected those to standard database searching using SpectroMine and again only included unique peptides consistently identified in three technical replicates at indicated peptide inputs. As expected, this data shows that, already at 10 ng total peptide input, DDA fails to provide consistent peptide identifications across multiple replicates (Fig. 3*C*). Further, across all inputs we observed a drastic reduction in peptide identifications of up to 50% when intersecting only two replicates for DDA analysis without data imputation (Fig. 3*D*). This is partially recovered in DIA data, despite total peptide identifications being generally much lower when compared with DDA data (Fig. 3*D*). In contrast to the standard peptide-based data analysis workflows, for both acquisition strategies, the ID-independent approach for DIA-TMT consistently yielded more than 30,000 datapoints across all replicates and peptide input (Fig. 3*D*). Our findings suggest that despite the reduction in peptide sequence overlap at ultralow input, the ID-independent data analysis strategy indeed also recovers quantitative data from ultralow abundant precursors. Additionally, the universal RT alignment and postprocessing strategy subsequently poses the chance of recovering peptide identification of sparse MS/MS scans and to characterize the cell type in detail.

### DIA-TMT Outperforms Standard DDA in the Characterization of Cell Types

**Fig. 4:**
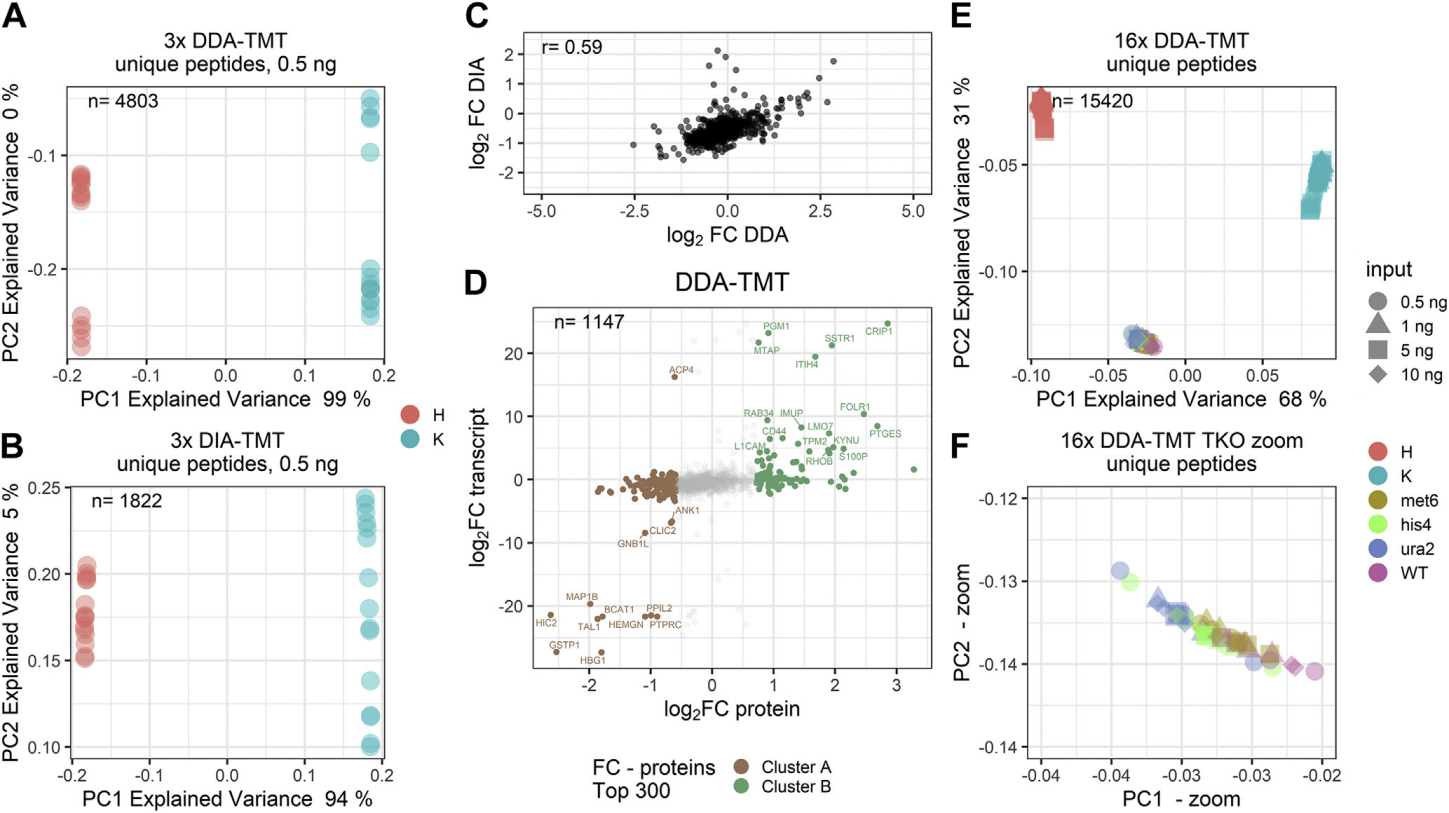
**Identification-independent data aggregation allows for the analysis of closely related cell types.** PCA of three analytical runs with standard peptide-based data aggregation at 0.5 ng total peptide input and missing value imputation of (*A*) DDA or (*B*) DIA acquisition schemes. n = number of unique peptides included in PCA. *C*, correlation of the average log_2_ fold change of proteins identified in DDA and DIA experiments displayed in Figures 3*A* and 4*C*. r = Pearson correlation estimate. *D*, intersection of transcriptome (FPKM) with DDA proteome data with top 300 proteins colored according to cluster contributions and top 60 proteins are labeled. n = protein identifications across all analytical runs after missing value imputation. PCA of 16 DDA runs based on peptide-based data aggregation with missing value imputation runs at four peptide inputs (0.5, 1, 5, 10 ng). *F*, zoom into TKO11 cluster displayed in panel *E*. Samples are colored according to channel loadings and the respective peptide input is indicated with different *symbols*. H = HeLa cells, K = K562 cells and the respective TKO11 strains (*i.e.*, WT, knockouts: *met6*, *his4*, *ura2*). n, number of unique peptides included in PCA.

Finally, to directly compare state of the art peptide-based DDA strategies to our ID-independent DIA-TMT data, we merged triplicates of HeLa and K562 mixes at 0.5 ng total peptide input and subjected them to standard database search using SpectroMine. Due to the strong reduction of datapoints available for postprocessing, we performed noise imputation for both peptide-based datasets, as described above. Although different numbers of data points (*i.e.*, DDA: 4810, DIA: 1822) were used for PCA, both unique peptide sets allowed a distinction between the two human cell lines *via* the first two PCs, even at 0.5 ng total peptide level (Fig. 4, *A* and *B*). Additionally, despite the slightly compressed fold changes in DIA compared with the DDA, quantification of common proteins positively correlates (Fig. 4, *C* and *D*). Importantly, like DIA-TMT data, the peptide-based analysis of ultralow input DDA data paralleled published RNAseq data of HeLa and K562 cells (Fig. 4*D*). However, despite noise imputation, the merged DDA dataset only yields 1147 protein groups to intersect with RNAseq data, which contrasts with 1707 protein groups for DIA-TMT data (Figs. 3*A* and 4*D*). This suggests that the peptide-based analysis of both DIA and DDA data recapitulates expected protein abundance and fold changes between cell lines (Figs. 3*A* and 4*D*).

**Supplemental Figure S2:**
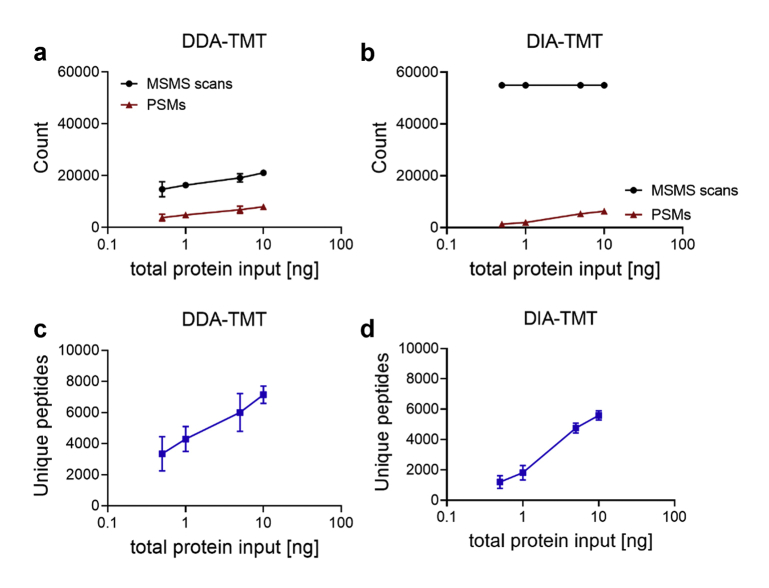
**Evaluation of identifications in DDA and DIA-TMT acquisition strategies.** Comparison of (*A*) DDA and (*B*) DIA experiments, showing the numbers of MS/MS scans (*black*), PSMs (*brown*), and unique peptides (*blue*) using (*C*) SpectroMine database search (DDA) or (*D*) Spectronaut library search (DIA) at various peptide input levels (*i.e.*, 0.5 ng, 1 ng, 5 ng, 10 ng). Mean and standard deviations are shown.

Further, despite higher identifications in DDA, the precursor stochasticity reduces replicate overlap to less than 40% of unique peptides identified per analytical run or requires computational generation of quantitative data. For example, at 0.5 ng total peptide input, we identified 1302 and 3843 PSMs or 1202 and 3348 unique peptides in the DIA and DDA acquisition modes, respectively (supplemental Fig. S2, *A*–*D*). Peptide-based data aggregation of three 0.5 ng total peptide input samples constrains the dataset to 1471 and 503 unique peptides for DIA and DDA data, respectively. The 10 ng total peptide input samples yielded 6308 and 8030 PSMs or 5598 and 7144 unique peptides from DIA and DDA data, respectively. After merging three replicates at 10 ng total peptide input, the dataset was reduced to 4371 or 5418 unique peptides DIA and DDA data, respectively (Fig. 3, *B* and *C*). This data indicates that despite higher numbers of initial peptide identifications in DDA, the majority are not identified across replicates. This contrasts with DIA-TMT where down to 500 pg per sample more than 70% of all unique peptide identifications are identified across multiple replicates without the need to computationally generate quantitative data (Fig. 3*C*).

Next, we assessed if peptide-based and noise-imputed DDA data would perform similarly to DIA-TMT data in defining and characterizing underrepresented cell types. For this, all 16 HeLa and K562 or TKO11 yeast datasets were merged and subjected to standard database search using SpectroMine. Peptide-based aggregation yielded only 18 peptides shared across all 16 analytical runs, which is partially due to the stochastic precursor sampling but again mainly a result of low sequence overlap between yeast and human. We therefore performed noise imputation and included all 15,423 peptide identifications in the PCA. Interestingly, similar to both, the ID-independent and peptide-based DIA-TMT analysis, the three main cell types cluster based on PC1 with 68% explained variance and the two cell types (*i.e.*, yeast and human) are separated on PC2 with 31% explained variance (Figs. 2, *C* and *E* and 4*E*). However, in contrast to ID-independent DIA-TMT analysis (Fig. 2*D*), the peptide-based DDA data does not separate the single protein knockouts (Fig. 4*F*). This shows that while peptide-based and noise-imputed datasets mostly recapitulate the expected cellular identity, in large homogeneous datasets, only the ID-independent DIA-TMT analysis successfully identifies single protein knockouts.

## Discussion

Taken together, we here demonstrate that ID-independent DIA is scalable and yields meaningful clusters of both, closely related cell types (HeLa *versus* K562), different composites of distinct species (human *versus* yeast), and even single protein knockout cell lines (TKO11 yeast). Our combination of isobaric multiplexing in DIA acquisition mode allows to generate comprehensive proteome signatures independent of sample origin or input level. We demonstrate that DIA in conjunction with our ID-independent data aggregation strategy averts the accumulation of “missing data” and retains quantitative data across multiple TMT batches without the need to impute computationally generated values (Figs. 1, *D* and *E* and 2, *B*–*F*). This is in stark contrast to the standard peptide-based method, which drastically reduces quantitative information even in combination with data imputation in the analysis of ultralow input samples (Fig. 4, *A* and *B*).

Our ID-independent multiplexed DIA anticipates coisolation, cofragmentation, or ratio compression and takes advantage of the unique sample profile generated through the conjunction of noise, background, and precursor ions. While ratio compression in general is a well-known drawback of RI-based quantification (even in DDA approaches with small isolation windows) (20, 25, 26) with numerous approaches to characterize and address this issue (22, 27, 28, 29, 30, 31, 32), we here show that the intentional coisolation of precursors in DIA does not impair sample classification. The uniform measurement of all ions irrespective of their origin (*i.e.*, sample or background) allows for a complete signature of the sample, reflecting on even slight expression changes between the samples.

Similarly, underrepresented cell populations and their identity can be identified postacquisition using our method, which is increasingly important when analyzing limited and complex biological samples other than homogeneous cell lines. The prospect of species- and cell-type-independent analysis will allow for studying diverse samples without *a priori* knowledge about the specimen. Even though the analysis of real single cells will expectedly dramatically increase noise and background ions, we are confident that multiplexed DIA will facilitate the generation of hypothesis-free single cell proteome signatures. Our novel DIA ID-independent analysis of large numbers and low concentration input samples might thus contribute to a universally applicable workflow for the study of protein expression across large cohorts.

## Data Availability

All mass-spectrometry-based proteomics data have been deposited to the ProteomeXchange Consortium *via* the PRIDE partner repository with the dataset identifier PXD023574. The conversion script to generate TMT libraries and all R-scripts are deposited *via* GitHub (ctorteckac/DIA-TMT). The reporter ions for DIA_TMT analysis within Spectronaut will only be visible from Spectronaut version 15 and higher. This in combination with the TMT library generator provided *via* GitHub allows to export the quantitative information from the report perspective. This workflow is not fully supported by Biognosys at this point. For questions or support, please contact support@biognosys.com or oliver.bernhardt@biognosys.com.

## Supplemental data

This article contains supplemental data.

## Conflict of interest

The authors declare that they have no conflicts of interest with the contents of this article.
